# “Fitting in whilst standing out”: Identity flexing strategies of professional British women of African, Asian, and Caribbean ethnicities

**DOI:** 10.3389/fsoc.2023.820975

**Published:** 2023-03-23

**Authors:** Victoria Opara, Michelle K. Ryan, Ruth Sealy, Christopher T. Begeny

**Affiliations:** ^1^Bath Business School, Bath Spa University, Bath, United Kingdom; ^2^Department of Psychology, University of Exeter, Exeter, United Kingdom; ^3^Global Institute for Women's Leadership, Australian National University, Canberra, ACT, Australia; ^4^University of Exeter Business School, Exeter, United Kingdom

**Keywords:** strategic identity flexing, intersectionality, racio-ethnicity, work organizations, gender, advantage, inequalities, women-employment

## Abstract

**Introduction:**

Professional British women of African, Asian, and Caribbean (AAC) ethnicities contend with unique challenges and experiences in the workplace. These challenges are often due to experiences that occur at the intersection of gender and ethnic identity, thus many professional white British women (of Anglo-Saxon decent), do not face the same challenges. AAC women are more often discriminated against, excluded from informal networks, and their contributions credited to someone else. We take an intersectional theoretical approach to better understand both the disadvantaged experiences and the possible advantaged experiences that British AAC women face, based on their experiences as AAC individuals, as women and as AAC women. The study seeks to 'give voice' to the experiences of AAC professional women, due to the limited amount of scholarship that adequately considers their workplace experiences. We consider the ways that their identity produces qualitatively different experiences determined by the context, by the nature of interpersonal encounters or by both the context and interpersonal encounters.

**Methods:**

We conduct real-time online written interviews and utilize thematic template analysis to understand whether AAC women strategically flex identity at work. We present four major themes. These are (1) the benefits of identity flexing, (2) the role of specific stereotypes, (3) context specific opportunities, and (4) the costs of identity flexing. We draw on literature that suggests that AAC women's identity experiences are not exclusively oppressive.

**Results:**

We find that unique experiences, occurring at the intersection of facets of identity may also yield forms of relative advantage, wherein individuals are able to adaptively leverage opportunities. Our results demonstrate that where the facets of one's identity are more varied, there might be more chance that the particular identity will be valued in a given social context. As a result, the individual may try to assimilate with certain identity groups (through flexing), as the context directs.

**Discussion:**

Nevertheless, engaging in identity flexing strategies has costs for women's well-being, such as needing to perform increased amounts of emotional labor and heightened feelings of frustration. Overall, these costs (as well as benefits), evidence the complex nature of identity flexing and the likely negative well-being implications that could ensue for the individual.

## Introduction

UK work organizations continue to be sites of inequity, group-based marginalization, and discrimination for many groups, such as those groups based on class, gender, racio-ethnicity, and religion (see Tillman, 2004; Burton and Chi Vu, 2020; Ryan et al., 2020). When we consider the forms of discrimination that African, Asian, and Caribbean (AAC) ethnic women, are likely to face, in the UK context, we know that racio-ethnic identity (i.e., identity based on racial and/or culturally and ethnically distinct groups, e.g., Cox and Nkomo, 1990) and gender prevent AAC women from participating fully in UK work organizations (Bell and Nkomo, 2001; Okechukwu et al., 2013; Sealy et al., 2018; Opara et al., 2020). British women of African, Asian, and Caribbean ethnicities (AAC) denote the UK's “non-white” population and comprise different racial and ethnic groups including African (14%), Arab (4%), Asian other (9%), Bangladeshi (5%), Black Caribbean (10%), Chinese (5%), Indian (20%), Mixed (8%), Pakistani (13%), and other (12%) (BITC, 2015). In total, this group comprises 14% of the UK's population, with those who are identified as white comprising 86% of the UK's population. The UK's white population includes white British individuals, those with white Eastern European backgrounds, white Irish, and those from white traveler backgrounds (GOV.UK, 2020).

The need to better understand and appropriately esteem British AAC women stems from their shared ethnic minority experience within the UK, despite the prevailing qualitative differences concerning how a particular ethnic minority experience will manifest against another (Opara et al., 2020). Discrimination within work organizations persists along racio-ethnic and gender lines, for AAC professional women (Rodriguez and Scurry, 2019; King et al., 2022). In turn, it renders the use of the well-known BAME (Black, Asian and minority ethnic) acronym redundant. The acronym BAME commonly used in the UK, has been found to be problematic (Sandhu, 2018). This is due to the BAME category comprising everyone in the UK that does not identify as white-British, so this would include all other white ethnicities including white Irish and white Roma individuals (Bhopal, 2004; Doldor and Atewologun, 2020). Eastwood (2021) also highlights that for some Black (African/Caribbean ethnic) individuals, use of the BAME acronym doesn't highlight the damaging impact of colorism, therefore, it does not capture the qualitatively distinct lived experience that occurs across racio-ethnic lines for many women in the UK.

Indeed the experiences of “non-white” individuals within the UK will differ to those experiences of white individuals, due to the forms of discrimination that African, Asian, and Caribbean (AAC) ethnic women exclusively, are likely to face (Cox and Nkomo, 1990; Noon and Ogbonna, 2021). Discrimination within work organizations, endures along racio-ethnic and gender lines, when we consider AAC professional women (Rodriguez and Scurry, 2019). This means that their experiences are important and need to be better understood. We did not deem the focus on a specific ethnic group such as “Asian ethnic women” or “African ethnic women” appropriate, in that this would unfairly disregard one group's experience over another; as though, the particular “flavor” of racio-ethnic discrimination is all that matters. Given the far-reaching effects of group-based discrimination, it is not surprising that scholars have called for research that considers the workplace experiences of women (see Fawcett Society Report, 2017; Financial Reporting Council, 2018; Begeny et al., 2020), or members of AAC ethnic groups (see Atewologun and Sealy, 2014; Opara et al., 2020), as well as research that considers those experiences which occur at the intersection of racio-ethnicity and gender (see Corlett and Mavin, 2014; Showunmi, 2018; Begeny et al., 2021).

Despite the push for a wider body of research, that considers the workplace experiences of AAC women, current research is lacking, as it focuses predominantly on experiences of disadvantage and oppression. This focus on disadvantage means we know less about the nature of the specific opportunities that AAC women may have, beyond those experiences of disadvantage and oppression. We say this in order to challenge a static dichotomous assumption that one is either advantaged or oppressed. This type of assumption propagates a static and singular identity narrative, which is problematic. As authors, we take a theoretical position that aligns with Holvino's (2010) intersectional perspective of simultaneity, from this, we come to understand that it is possible for one to experience both disadvantage and advantage simultaneously, as it is possible for an individual to be an oppressor, a member of an oppressed group, or concurrently oppressor and oppressed (Collins, 2000; Dhamoon, 2011). An identity which is found to be unique within the respective social context (Parent et al., 2013), will likely inform some distinct avenues of advantage (context dependent, e.g., at home, at work, national context, international contexts and this includes the contexts of discriminatory and bias systems). This means that AAC women, should not be considered simply as recipients of discrimination and racist treatment, they also respond to and shape workplace experiences in agentic ways (Atewologun et al., 2017). This is not to suggest that AAC women are not subject to structural constraints, such as systemic forms of discrimination and marginalization, rather it emphasizes that more attention ought to be given to AAC women's responses to such discriminatory treatments and experiences beyond discrimination and marginalization. We recognize that the experiences of AAC women certainly encompass experiences of disadvantage, marginalization, and discrimination (and we in no way seek to trivialize or disregard these experiences). Nevertheless, these types of occurrences do not capture the sum of AAC women's professional experiences.

To a large extent, current understanding about AAC women and Black women specifically, is informed predominantly by the experiences of Black US women (Johnson and Thomas, 2012). Whereas it is the particular positionality of an AAC woman within her UK context and relative to the interpersonal exchanges at work, that directs a more fluid and transient evaluation of her organizational experiences (Hwang and Beauregard, 2021). For instance, a woman in the UK, may experience the legacy effects of disparate underpinning systems of colonialism, when compared to a woman in the US, who may experience the effects of both colonialism and slavery/indentured servitude. Equally, a woman in South Africa would be subject to the legacy effects of apartheid, but this would not be the case for UK and US based AAC women (Carrim and Nkomo, 2016). An ability to recognize this inherent difference is needed, to prevent all AAC women being clustered together in a monolithic way. The application of intersectional research can indeed become limiting if national context and underpinning systems of difference, domination and oppression are not regarded (Dhamoon, 2019; Opara et al., 2020).

Understanding of the unique experiences that occur at the intersection of gender and ethnic identity within different social spaces, is incomplete. It means we are presently unable to answer the question: *Do the unique workplace experiences of AAC professional women comprise forms of relative advantage?* Attempting to address this gap, a small body of research that considers the identity experiences of AAC individuals and the ways that they negotiate their identity, has emerged in the fields of diversity and inequality scholarship, organizational studies, social and organizational psychology (see Purdie-Vaughns and Eibach, 2008; Cole, 2009; Showunmi et al., 2015; Opara et al., 2021). Identity flexing adopts a *strength-based* approach to the study of racial and ethnic minority relations, rather than a *deficit-based* approach, due to, identity flexing strategies epitomizing one's determination to be understood based on the strengths, knowledge, creativity, adaptiveness and insights that are implicit to one's identity, be that AAC women or any other intersectional identity (e.g., invisible minorities, Muslim women, AAC men).

Regarding or highlighting the existence and use of identity flexing strategies, does not suggest that AAC professional women do not also experience myriad forms of bias and discrimination. However, what it does do, is make efforts to move past a long-standing narrative in the (predominantly white and Western) research literature that portrays AAC women largely as passive victims—as *primarily* a target of bias and discrimination, which is in many ways is quite dehumanizing and discriminatory.

In this paper we will examine whether British AAC women are able to “flex” identity in a strategic or constructive manner, and to what extent the use of identity flexing strategies, allows women to leverage relative advantages. The study research questions are:

“*Do British AAC women flex their identity in a strategic way, within the workplace?*” And if so, “*what are the outcomes that result for these women?”*

To explore these questions, we draw on the literature about intersectional identity work and identity management strategies, AAC women and women's organizational experiences to highlight the complex and multifaceted nature of how identity flexing may occur within AAC women's workplace experiences. To the best of our knowledge, this is one of the first studies that considers identity flexing as a *resource-based* approach—that emphasizes strategic opportunities or experiences of advantage for British AAC professional women, in the process of self-identification and identity construction.

### Identity and intersectional identity work

Within the notion of identity and identification exists a discourse that concerns what it means to incorporate efforts to manage one's physical appearance, as well as particular behaviors and verbal manifestations of identity (Begeny et al., 2022b). This is due to identity invoking the question of “who am I?” and also “how am I to act?” (Knights and Clarke, 2017), contrary to identity assumptions that infer unitary, static, or persisting continuity (Maylor, 2009). These identity discourses involve notions of manifold and dynamic selves (Ibarra, 1999; Knights and Clarke, 2017). There is potential to experience one's self and self-identity as unique, inherent and dynamic points of coordination for identity in the context of work (Petriglieri and Stein, 2012; Atewologun et al., 2017). These inherent and dynamic aspects of understanding identity are referred to as “identity work.” Identity work has been defined as “*the ongoing mental activity that an individual undertakes in constructing an understanding of the self, that is coherent, distinct and positively valued”* (Alvesson et al., 2008, p. 15). The concept of “identity work” is key to understanding how identity is chosen by the individual and/or ascribed to individuals (Brown, 2015). From an identity work perspective, *identity is seen as something done, rather than a static category*. This may take the form of “covering,” that is to substitute one social identity category for another, or “redefining,” that is to develop ones “own unique set of values and goals based on a positive sense of (AAC) identity” (amongst other forms of identity work) (Slay and Smith, 2011).

There is a small amount of intersectional identity work scholarship that helps to advance knowledge, and appropriately theorize the juxtaposition of disadvantage and advantage (for an example, see Atewologun et al., 2017). This theorizing falls within the realm of [several lines of theory and research on] intersectionality. Therefore, a focus on intersectional identity work provides the much-needed framework to understand the strategies that women with AAC identity might adopt in response to identity incongruence. However, a focus on intersectional identity work alone is not sufficient when seeking to embed identity experiences of racio-ethnicity and gender, within structural and institutional systems of disadvantage and privilege. Undoubtedly, a focus on intersectional identity work alone does not explicate the full nature of identity experiences within work organizations, neither does it allow us to understand the contextual nuances relative to structural and institutional systems of disadvantage and privilege. For instance, AAC woman and the nature of their experiences manifests in unique ways, within variant systems of difference, from one social context to another. Dhamoon (2011, p. 234) defines systems as “*historically constituted structures of domination such as racism, colonialism, patriarchy, sexism, capitalism and so on.”* Therefore, it will not always be effective, to focus only on identity, but rather on what the interaction between identity and the contextually instituted system reveals about an experience (Dhamoon, 2011). In addition, intersectional identity work seldom considers identity construction, from a *resource-based* perspective. Much of the current intersectional identity work scholarship does not fully regard how intersectional identity experiences manifest for AAC women, and from a *resource-based* approach—an approach that considers moving intersectional identity work knowledge beyond forms of oppression (see Corlett and Mavin, 2014; Carrim and Nkomo, 2016). In addition to this, few of these studies are considered within the context of UK organizations (for one example, see Atewologun et al., 2017). The need to consider how British professional AAC women particularly, make use of identity flexing strategies is necessary to expand existing knowledge.

### Identity management strategies of professional AAC women

Identity flexing may allow AAC professional women to explore different aspects of their self-concept and in turn facilitate improved interactions with individuals belonging to different social identity groups (Begeny et al., 2022a). Self-concept, also referred to as self-image or self-identity, has been defined as, “the totality of an individual's thoughts and feelings with reference to themself as an object” (Jeong and Ko, 2020, p. 513). It is likely that AAC women will use different identity management strategies in the workplace, such as code switching (McCluney et al., 2021), identity shifting (Shih et al., 2010; Jackson and Sanyal, 2019) or identity flexing (Opara et al., 2020). Use of an identity management strategy should theoretically help AAC women exploit any opportunities that come from intersectional invisibility (Purdie-Vaughns and Eibach, 2008), such as others not knowing what behaviors to expect from them, or others being ignorant of their ethno-cultural norms (McCluney and Rabelo, 2018; McCluney et al., 2021). Jackson (2002) states that to reduce their susceptibility to stereotypical expectations and discriminatory actions, ethnic “minority” women may alter their actions or language to fit within a given environment. This process is known as identity shifting (see Jackson and Sanyal, 2019; Dickens et al., 2020).

Identity shifting, also known as identity negotiation refers to the conscious and unconscious process of shifting one's worldview and cultural behaviors in intercultural encounters (Dickens et al., 2019). Identity shifting tends to focus on a singular identity category at a time, such as erasing race, or silencing gender (see Dickens et al., 2019). A similar concept is code switching, this is a type of identity shifting that denotes the process of fluctuating between two or more dialects/languages (Crawford, 2021; McCluney et al., 2021). Identity shifting, refers to a broader set of alterations (than code switching), such as: attire, dialect, language, behavior, hairstyles, morals, and values (Shih et al., 2013; Dickens et al., 2019, 2020). In general, the focus on a single facet of identity (e.g., gender or race), is limiting, as research has shown that social identity categories do not operate independently of themselves, but they interact and intersect, creating an entirely unique identity experience at the intersection (Crenshaw, 1991; see also Kang and Bodenhausen, 2015).

In contrast to identity shifting, identity flexing' (Opara et al., 2020), refers to the deemphasizing of more than one negatively valued aspect of identity and the emphasizing of more than one positively regarded identity facet. Identity flexing also regards the interaction between identity experiences at the intersection and contextually instituted systems of domination (i.e., sexism, capitalism, colonialism, apartheid, racism, patriarchy), which will also inform those who are *dominant* and the *dominated* in any given social context. From the above, we can infer that identity flexing is unlikely to occur exclusively among AAC women. Many people are at the intersection of diverse (undervalued) facets of identity, and these individuals may also engage in identity flexing strategies, e.g., biracial individuals, working class individuals, individuals with disabilities, invisible minorities, AAC men. We believe that AAC women's specific identity is intrinsically as valuable as any other identity (e.g., AAC men, white men, white women), and are not inherently devaluing. Nonetheless, it is the treatment of others toward AAC professional women, that speaks to the undervaluing of their identity. The study focus' is on the lived experiences of AAC women, due to the shortage of scholarship that adequately considers the multifaceted nature of AAC professional women's workplace experiences from a strength-based perspective. To mitigate increased negative stereotyping and scrutiny, AAC women may be moved to behave in ways that reduce their distinctiveness and increase perceived belonging (Dickens et al., 2019). This could occur relative to other AAC individuals (in contexts where AAC individuals constitute the numerical majority). However, within UK work organizations, it is more likely to occur in relation to white British individuals, due to UK work organizations being predominantly and prototypically white British work environments (Atewologun and Sealy, 2014).

In this sense the authors propose that flexing of identity can be considered a *resource* or a source of strength. However, the pressure to adjust identity in this way, particularly in the workplace, can be onerous (Shih et al., 2013). For instance, the need to flex identity may trigger internal conflict and may also contribute to distorted perceptions of the self, this we say based on work that considers identity shifting strategies among Black women, in the workplace (see Purdie-Vaughns and Eibach, 2008; Dickens and Chavez, 2018; Dickens et al., 2020). This means that there might be a very thin line between identity flexing strategies being arduous and where identity flexing becomes advantageous and can be used strategically. As Bottomley (1991) argues, ethnic identity can be strategic and positional. This gives credence to the thesis that an identity can (in a particular context) be strategic and thus *used* strategically. Therefore, identity flexing could be considered the expression of an irreducible “otherness,” which is operationalized for the critical speaking position or contextual advantages that it offers (see Bottomley, 1991; Shih et al., 2013). As Bottomley (1991) asserts that identity can be regarded as strategic, in-line with Dickens et al. (2020), we suggest that AAC women may have access to more “strategies” when considering how to influence a situation in their favor, due to their multifaceted identity. For example, one could strategically flex identity to alleviate the consequences of discrimination as well as to capitalize on opportunities, based on identity, which may likely be positively regarded within certain inter-personal encounters (e.g., encounters with other AAC individuals, that hold positions of power) (Hwang and Beauregard, 2021).

Another example pertains to the non-prototypical nature of British AAC women's identity experiences, as they comprise a numerical minority, they are likely to negotiate their race and gender at higher frequencies than their white male counterparts (Castro and Collins, 2021). AAC women might then be better equipped to relate with people with multifaceted identity and less likely to fear or be threatened by difference (Leaf and Ngo, 2020). AAC women tend also, to have more diversity in their subjective intragroup networks (Mannix and Neale, 2005; Leaf and Ngo, 2020; Saak et al., 2021). This brings advantages based on identity, these advantages include greater cultural intelligence, wider reaching social networks and greater ability to navigate diverse social contexts (Roccas et al., 2008). An intersectional approach is important when examining the manifold identity experiences of AAC women in work contexts, as it causes us to question, who *needs* to flex identity? who *is able* to flex identity? And for what reason would one flex identity?

### Intersectionality and strategic identity flexing strategies

Intersectionality is rooted in African American feminist scholarship. Through Crenshaw's (1991) thesis, we are able to regard the ways in which race and gender mutually construct and reinforce each other. In other words, the notion that aspects of identity such as race and gender do not operate independently but rather coalesce to inform individual's lived experiences, has become a widely accepted notion. When Crenshaw (1991) coined the term intersectionality, she was seeking to confront the systems in which multiple oppressions were formed and experienced. We recognize that the meanings of multiple oppression are contextual and that intersectional experiences are historically contingent (Davis, 2008, p. 68). Furthermore, we understand that multiple oppressions are only part of the story, as what it means to be an AAC woman and the nature of the experiences that manifest at the intersection of diverse aspects of identity spans far beyond that of multiple oppressions alone. This is partially due to the variation in systems of difference or domination from one social context to another. Dhamoon (2011, p. 234) defines systems as “*historically constituted structures of domination such as racism, colonialism, patriarchy, sexism, capitalism and so on.”* The focus here is not necessarily on the identity itself, but rather on what the interaction between identity and/or contextually instituted system reveals about power (Holvino, 2010; Dhamoon, 2011). The interaction of diverse facets of identity and contextually instituted systems of domination will produce the dominant and the dominated within all societies across the globe. It means that it is possible for an individual to be an oppressor, a member of an oppressed group, or simultaneously oppressor and oppressed (Collins, 2000; Dhamoon, 2011). This perspective aligns with Tatli and Özbilgin's (2012) proposition that privilege and inequality are fluid and variable, contingent upon the situation in which the power dynamic unfolds. Intersectional analysis of power dynamics and systems of domination should therefore always be regarded when we consider who gets to strategically flex identity and in which ways can identity be flexed. For example, in the UK context, information about an AAC woman's nationality or religion (e.g., British, Christian), could become a resource in fostering connectedness and allowing her to identify with others, who also identify as Christian or British but may not possess the same ethnic, national, or gender identity as her (e.g., White British men, white British women, non-British AAC individuals—that is those AAC individuals born and socialized outside of the UK context, but have since relocated to the UK).

Highlighting that multiple oppressions are only part of the intersectional identity story for AAC women, denotes an effort to better understand the irrefutable strengths and insights that AAC women have. This is not to imply that things “aren't that bad” for AAC women. Instead, it is an effort to advance beyond a long-standing narrative that portrays AAC women as *largely* targets of discrimination (King, 2003; Ponce de Leon and Rosette, 2022). It is equally important to recognize that alongside this shift toward a greater recognition of AAC women's strengths, knowledge, creativity and insights, individuals who currently and have historically occupied positions of power, including white men, must actively work to address the multitude forms of bias and discrimination that they themselves have developed and regularly perpetuate. Historically and to date, AAC women have endured the burden of addressing these long-standing inequities.

Previous literature supports the notion that identity negotiation (the process through which people reach agreements regarding “who is who” in their relationships), and similarly identity flexing amongst AAC women is multidimensional, comprising of both advantages and disadvantages. Previous research has shown that (de)valuing of any social identity is a fluid notion, that fluctuates across space (social contexts) and time (social norms and trends) (Dhamoon, 2011; Tatli and Özbilgin, 2012). These differences in experiences are why intersectional research and intersectional theory has become popularized in the organizational and psychology fields (Dennissen et al., 2020), that is to regard the qualitatively different experiences that occur for individuals based on their social identity group memberships.

#### Problematizing power and privilege

Differences in power have implications for the nature of individual, organizational/structural, and societal experiences that one has (Rodriguez and Scurry, 2019). It is not possible to regard one's individual experiences of identity without regarding their structural/organizational experiences relative to the power relations of other identity groups such as white women and, AAC men (Showunmi et al., 2015). This is to say that, in some cases AAC women are likely to be flexing identity as a defense mechanism to buffer the ill effects of white privilege. Within Showunmi's (2018) reflection on the role of whiteness and intersectionality in comprehending Black (African/Caribbean ethnic) women's experiences in academia, we come to know that white privilege is like any social phenomenon, it is complex and in a white-supremacist society (such as the UK), all white individuals have some form of privilege, in some settings. White supremacy is a societal system that those with white privilege are born into (Eastwood, 2021). According to Eastwood (2021), a white-supremacist society, is any society, wherein a white majority frequently marginalizes “non-white” populations within both society and organizations, purely based on an individuals' distinct racial identity within the society. By definition, we can consider the UK to be fundamentally, a white-supremacist society, that was founded on these white supremacist ideals (Christian, 2016). We understand that our use of language may be uneasy for some, but we should always be open to change in our use of language, specifically when those changes can be used to highlight and or expose the ways in which certain groups continue to hoard and abuse power (Eastwood, 2021). This is not to say that all white individuals have equal amounts of privilege,^1^ but there are general patterns. Such privilege can be diminished and/or heightened, determined by the social context and other aspects of one's identity (e.g., gender, class, geographical location, level of education, accent) (Dhamoon, 2011).

When we consider the ways in which British AAC professional women utilize identity flexing strategies, there is also a need to consider power-related aspects of social group memberships, so not to skew our understanding of identity flexing amongst this population. This is especially important, as one cannot disentangle him or herself from the power-related aspects of social group memberships, this means that we are either directly benefitting from it or directly being injured by it. We say this to situate identity flexing within the broader context of power and advantage and expose identity flexing to be a strategy employed in the struggle for liberation (Dhamoon, 2019).

### The costs and benefits of identity flexing strategies

The pressure to regularly negotiate one's identity through identity flexing is likely to be burdensome for AAC women, in certain aspects. The need to flex one's identity is likely to lead to an internal sense of conflict, whereby one is left grappling with a skewed self-perception (Opara et al., 2020). When one must engage repeatedly in identity management strategies, this may become a source of stress and frustration (Sojo et al., 2016). Understanding the need of AAC professional women to flex their identity in work organizations, may help us to have a holistic understanding of their workplace experiences. Bell and Nkomo (2001) assert that in work organizations AAC women experience the ‘sticky floor' barrier posed by experiences of racism and sexism, and intersectional experiences comprising the interplays of raced and gendered experiences. We suggest that the flexing of identity may allow AAC women to experience more “advantageous” organizational experiences such as evading the sticky floor, by complying to the more acceptable prototypically white British workplace culture; but that this is unlikely to occur without costs. As, in accordance with Shih et al. (2013), the long-term use of identity management strategies, such as code-switching, identity shifting and identity flexing, may cause an unstable sense of self, and this can be linked to reduced psychological wellbeing (Shih et al., 2013).

Nevertheless, identity flexing can also be considered beneficial (Dickens and Chavez, 2018), in that individuals who have more diverse facets of identity or different kinds of identity categories are able to identify in many ways, within social situations, which has been shown to have positive implications for psychological wellbeing (Roccas et al., 2008). For instance, when the UK voted a decision in favor of “BREXIT,” to leave the European Union, this was a decision that led to a great sense of cynicism and distrust of Britons, particularly from European nationals working in the UK. This was also a time, whereby British AAC professional individuals got to “fly under the radar,” they could very easily distance themselves from the British identity at work, in a way that “white” Britons were not able to do. Given these potential costs and benefits of engagement in identity flexing amongst AAC professional women, the current study will explore the extent to which identity flexing can be taken as an advantageous strategy for AAC women to engage in, or whether it is disadvantageous for them, particularly when we consider their wellbeing outcomes.

## Method

We engaged a qualitative method and collected data through semi-structured online written interviews to investigate the specifics of participants' workplace experiences (see Opara et al., 2020, 2021; Hwang and Beauregard, 2021). The subtle realist philosophical position provided scope for emergent knowledge to be discovered and understood (Opara et al., 2021). This epistemological approach to knowledge, takes the position that knowledge is co-constructed. Therefore, within all social contexts knowledge of reality can be known in that, the phenomena remain independent, but the knowledge of them is co-constructed by the investigator and the investigated, and their independently existing reality. In accordance with Hammersley (2008), knowledge can be sensed or perceived *via* a direct means of investigation. This research design will provide opportunity for exploration (emergent knowledge), as opposed to operating by testing *a priori* hypotheses. We deem this to be the appropriate philosophical stance, given the personal nature of the research constructs and the underpinning epistemological belief that knowledge of reality can be known from one's personal perspective of it.

### Sample

Thirty British professional AAC women, all of whom were resident within England (see Table 1 below), were interviewed about how and when they flex their identity within work organizations.

**Table 1:** Biographical data of participants.

**Pseudonym**	**Age**	**Highest qualification**	**Job sector**	**Self-ascribed ethnic identity**
P1	25	Undergraduate degree	Human resources	Mixed Black Caribbean
P2	32	Postgraduate degree	Property and construction	African
P3	37	Postgraduate degree	Accounting and finance	Asian
P4	31	Undergraduate degree	Healthcare	Mixed Black-Caribbean
P5	28	Postgraduate degree	Consultancy	Black-British
P6	29	Postgraduate degree	Social care	British Asian
P7	40	Postgraduate degree	Civil service	Black
P8	46	Postgraduate degree x2	Food safety and agriculture	British Indo (Goa) Portuguese
P9	25	Undergraduate degree	Civil service	Afro-Caribbean
P10	64	Postgraduate degree	Social care	Chinese
P11	33	Postgraduate degree	Education	Mixed Black Caribbean and White British
P12	39	Postgraduate degree	Creative arts	British Born Nigerian
P13	39	Doctorate degree	Leadership/advisory	Mixed Indian Jamaican
P14	40	Doctorate degree	Education	South Asian, Sri Lankan
P15	32	Doctorate degree	Education (psychologist)	Black British Caribbean
P16	34	Doctorate degree	Criminology and sociology	Black Caribbean- I am not British, I don't even know what that means, even though I was born here
P17	39	Postgraduate degree	Healthcare	Black British African
P18	39	Undergraduate degree	Healthcare	Black British/Nigerian
P19	44	Postgraduate degree	Education (freelance)	Black British Caribbean
P20	25	Undergraduate degree	Healthcare	African
P21	45	Doctorate degree	Education	Black British (African)
P22	33	Postgraduate degree	Civil service	Black-British
P23	29	Doctorate degree	Education (psychologist)	Black-British
P24	30	Undergraduate degree	Digital marketing	Black Caribbean
P25	47	Undergraduate degree	Trade union	Mixed White and Asian
P26	30	Postgraduate degree	Education	British Chinese
P27	42	Undergraduate degree	Social care	Mixed Black-Caribbean
P28	33	Doctorate degree	Education	Nigerian (British Born)
P29	31	Postgraduate degree	Consultancy	Black African–British Born
P30	27	Postgraduate degree	Law	British of Indian origin

We considered a sample of *N* = 30 to be appropriate as we interviewed participants until we reached data saturation. In accordance with Hennink et al. (2017), saturation point should be used as a way of assessing when we had “heard it all” as well as when we had “understood it all,” this resulted in 30 interviews being conducted in total. The participants ranged from age 24–64 years of age. The job sectors represented included legal, academic, health, finance, creative industries, business consultancy, logistics, human resource management and both educational and forensic psychology. Women described their own racio-ethnic identity, according to how they identify relative to their racio-ethnic identity. Furthermore, we defined “professional” as an organizational position that involves a minimum of a bachelor's degree (or its equivalent) to practice (Opara et al., 2020). We used a combination of purposive and snowball sampling technique (Gioia et al., 2012; Woodley and Lockard, 2016). In the first instance, 17 British Professional AAC women were contacted and selected to take part in the study, by using the personal and professional networks of the authors, as well as reaching out to participants from a previous study. The women were recruited through online platforms such as Prolific Academic and Facebook as well as through reaching out to them *via* email. The initial 17 participants were then asked to refer any eligible contacts that they had. Those contacts were then contacted *via* email and this process continued until the desired sample size of *N* = 30 was achieved.

### Data collection

We conducted semi-structured written interviews with the women, consisting of 21 open-ended questions (see Appendix 1 in Supplementary material). Interview accounts were gathered using an innovative online written interview methodology realized through the Google Docs online platform (Opara et al., 2020, 2021; Banini and Tabai, 2022). We used online written interviews as they facilitate access to individuals (e.g., due to distance, sensitivity of the subject) and allow for a greater sense of anonymity and privacy. This is due to both the interviewer and interview participant being in different settings, meaning concerns of bias between interviewer and participant are reduced, permitting for an increased sense of anonymity, in that even the interviewer was unable to see the interviewee. Nonetheless, we deemed this approach suitable in order to answer the research questions of: “*Do British AAC women flex their identity in the workplace, in a strategic way?*” And if so, “*what are the outcomes that result from this strategic identity flexing?”* The interviews were synchronous, that is both the interviewer and interviewee engaged with the document at the same time, in real time. The author who conducted the interviews took the position of outsider when conducting all interviews, in that she was able to listen to participants without influencing or contributing to the responses that participants gave. This was possible due to the nature of written interviews, whereby the interviews were not able to see nor hear the interviewer. This being said, the written interviews still allowed for a good level of communication between the interviewer and interviewees, due to interviews being synchronous. We recognize that in-person, face-to-face interviews are deemed advantageous when seeking to build trust between the interviewer and interviewee, and when compared to written interviews. Nonetheless, the study's research design provided alternative opportunity to build trust using this particular method, as we were able to call participants prior to participating in the interview. The phone call allowed the interviewer to brief participants. This meant that the preliminary conversation prior to conducting the interview gave opportunity for rapport to be built and trust to be fostered.

Interviews lasted ~1.5 h, with each interview comprising on average, ~2,000 words. In actual fact, the written interviews allowed for a greater sense of privacy, without detracting from the need of the interviewer to remain open to the interviewee, especially when discussing sensitive topics such as experiences of race and gender. All the interviews were transcribed in English as all women were professionals, living and working within England, thus all women have strong command of the English language. Interviews took place over the course of two months from the month of April–June.

During interviews we explored how participants negotiated their identity experiences in the workplace. Particularly, we considered whether participants were able to flex particular facets of their identity in such a way that could produce forms of advantage. Questions explored whether or not it was possible to modify behavior or play up a certain identity to capitalize on the level of advantage or opportunity, whether it was ever possible to use aspects of one's ethnicity/race and/or nationality to gain an advantage or navigate a situation. Interviewees were also asked about their ability to be authentic in the workplace, that is whether or not it was possible to bring their “whole selves” to work. All interview questions were followed up with probing questions (see Appendix 2 in Supplementary material).

The first author (who identifies as British born, Nigerian-Igbo indigene) conducted all of the interviews, the identity of the interviewer as a professional AAC woman was disclosed during the initial stages of the interview. Furthermore, due to the author having an indigenous Igbo surname, this proxied her racio-ethnic identity, for those who were able to recognize the name's origin. Nonetheless, we did not deem this a weakness in the study's design, but rather a strength in that the subtle realist epistemological approach asserts that, there is a single reality (not multiple realities corresponding to different perspectives); yet obtaining knowledge of this reality, must be drawn from both the researched and the researcher. Hammersley (2008) argues that, the researcher is part of this single reality, not separate from or above it. In other words, the predeterminations of the researcher (i.e., researcher bias) can provide insight and novel perspectives, rather than simply being a source of error. Nonetheless, we were mindful of remaining open, and we were sure to welcome any information that we didn't expect to find (Miles and Huberman, 1984). In addition to this, all authors regularly discussed the data collection, to ensure a reliable and robust interview process.

### Data analysis

As the study's research design is informed by previous research (Opara et al., 2021) through the interview data we aimed to provide context to the outcome data of that study. Thus, there were a number of *a priori* (deductive) codes taken from the interview protocol. These include strategic identity, identity flexing, wellbeing, and authenticity. Undertaking a thematic template analysis (Brooks et al., 2015), we engaged in both deductive and inductive coding. A deductive approach was taken early in the analysis process, as analysis in this study was based on findings from a past study (see Opara et al., 2020). This meant that the first-order codes of strategic identity, identity flexing, wellbeing, and authenticity, were based on previous literature. In-order to expand knowledge beyond what we already knew, an inductive approach was taken to generate all subsequent codes and themes through open (unrestricted) coding, followed by the finessing of themes.

In many cases, it is necessary to employ a combined approach when the study has particular issues that it seeks to explore upfront, but also seeks to leave room for deeper exploration of these issues, relative to the way participants assign meaning to these phenomena. The authors consulted frequently to ensure that the inductive codes that were generated adequately represented a particular construct or concept. We then organized the initial codes into clusters (see Table 2; Brooks et al., 2015).

**Table 2:** Initial codes, emergent clusters and final themes.

***“I lean in to the identity that highlights the desired side of me, that way I get to control the narrative of me”* (Black British Caribbean: Education Consultant)**	**• Controlling the narrative**	**Empowerment through identity flexing**	**The benefits of identity flexing**
“*I definitely push forward my blackness and Africanness, it's not always a negative, it depends on who you are coming across.”* (**British Born Nigerian: Retail Business Owner**)	• Versatility in assimilating to the salient identity		
“*I don't feel like I need to modify my behavior to gain opportunities. Being myself has allowed me to form strong working relationships. The nurses seem to appreciate my work. I now work with predominantly more African nurses.”* (**African: Mental Health Care Nurse)**	• Flexing identity to build professional relationships	Identity flexing leading to organizational influence	
“*I am considered for more leadership roles …] I can say I am a woman, I am brown or black, so you can tick a few boxes with me”* **(British-Goa, Indo Portuguese origin: Technical Consultant)**	• Flexing identity for professional advancement		
“*I am now authentically and unapologetically myself”* **(Mixed Black and Indian Caribbean: Health Administrator)**	• Unapologetically myself	Intersectionality, stereotypes and identity flexing	**The role of specific stereotypes**
“*I am honest when I can be and explain that “Black” is not a homogenous group and all of our experiences are not uniform. This is easy for me to do as an African woman”* **(Black British (African) /Senior Lecturer in Law)**	• Less backlash for being an agentic (AAC) woman		
“*I get away with playing up certain identities at work, it can often work in my favor”* **(Mixed Black and Asian Caribbean: Human Resources Manager)**	• Strategic identity flexing	Contextual advantage	**Context specific opportunities**
“*I highlight my gender at work. I act very feminine especially to “white” management, they receive me better that way”* **(Black British Caribbean: Education Consultant)**	• Modification of behavior from home to work		
	• Modification of behavior according to the social context		
“*I can't be 100% myself in the workplace”* (**Afro Caribbean: Query Management Leader)**	• (In)authenticity	Identity flexing leading to inauthenticity and increased emotional labor	**The costs of identity flexing**
“*One can minimize weaknesses by playing to strengths, but this requires an investment of energy and it begins to wear you down*” **(Mixed Indian and Black Jamaican: Social Psychologist and Leadership Strategist)**	• Wellbeing		
• Less social direction			

We then reviewed the clusters to ensure that the data within each cluster aligned in a theoretically meaningful way, confirming clear distinction between respective clusters. We then organized the clusters into their final four major themes, presented below in Table 2.

## Results

Participants' workplace experiences of strategic identity flexing demonstrated multiple benefits and advantages perceived by the individuals. However, participants also reported experiences of disadvantage regarding the inability to be authentic in the workplace, which in turn had negative implications for their wellbeing. We define four major study themes: (1) the benefits of identity flexing, (2) the role of specific stereotypes, (3) context specific opportunities, and (4) the costs of identity flexing. Our results demonstrate that individuals who possess diverse facets of identity, of which some may be negatively stereotyped, may strategically flex or highlight other valued facets of their identity within social contexts.

### Theme 1: The benefits of identity flexing

For our participants, their diverse identity generated unique workplace experiences that at times were perceived as advantageous. The women reported that flexing their identity as a British AAC woman, at times allowed them to leverage certain advantages.

#### Controlling the narrative

Participant 15 described how her social group memberships give her greater opportunity to navigate certain workplace contexts, due to her ability to “control” the specific narratives that her work colleagues may form of her:

“*Having my social identity categories can feel advantageous as I feel that to a degree, I am more able to control people's narrative of me, as they are less likely to know what exactly to expect with me, and so I consider myself to be quite flexible.”* (**Participant 15—Black British Caribbean: Educational Psychologist**).

This participant makes the point that her identity could be deemed strong, in the sense that others were not aware of what to expect. In this work situation this participant experienced a heightened sense of efficacy, owing to her distinctiveness within the workplace setting. It is suggested by this participant, that this is a form of advantage, as being able to control the narrative, is linked to greater versatility at the interpersonal level. This is because of the prevailing ignorance concerning what types of behaviors to expect from British AAC women. As stated earlier, Purdie-Vaughns and Eibach (2008), refer to this as *intersectional invisibility*, referring to a mix of advantages and disadvantages stemming from the non-prototypicality of AAC women in the UK, meaning that others are not so readily able to label them or “pin them down” with a particular set of preconceived notions/stereotypes. While this perceived ability to control the narrative may be partially linked to intersectional invisibility, it is also linked to AAC women themselves being more accepting of ambiguity and uncertainty.

“*I am perhaps more at ease with being uncomfortable within an interaction as it is not an unusual circumstance for me and I feel that it perhaps makes me more aware of how others might only present parts of themselves, and why… even if they don't admit this, I am more able to sense when they are doing this”* (**Participant 17—Black British African: Pharmacist**).

In other words, AAC women's experiences provide them with greater awareness of needing to navigate ambiguity and uncertainty, and what this might look like. In some situations, this could be viewed as positive, as the women were able to ascertain when others were possibly grappling with ambiguity and only presenting a part of themselves. Use of identity flexing allowed the women to be at greater ease with uncertainty, as they can modify behaviors in real-time in-order to mitigate any ill-effects of being a recipient of negative stereotyping. By flexing identity in this way, women are able to control the narrative of others concerning them.

#### Versatility in assimilating to the salient identity

The degree to which individuals were aware of their greater ease with ambiguity and their ability to adapt to situations, afforded them a greater sense of versatility and allowed them to flex their identity to gain beneficial outcomes more readily. For instance, a retail business owner, who identifies as British Born Nigerian (**Participant 12—British Born Nigerian: Retail Business Owner**), spoke of how she sometimes “*pushes forward her Blackness or Africanness.”* Here she was referring to wearing clothing items that proxied “Black” culture, such as head wraps or using dialectal speech to signal her ethno-cultural heritage. She explained how she is able to flex her identity and adapt to a situation once presented with the appropriate stimuli to do so. When presented with a situation which involved an individual with a shared racio-ethnic identity, or intersectional identity, the participant often uses terms that signal her African ethnicity. For example, using the term “*oyibo”* or “*obroni”* mid-sentence, which at its simplest form means white person across a number of West African nations. In another example, a participant made her Britishness salient when interacting with a white British colleague, to “*clarify that I belong here (in the UK)”* [**Participant 21—Black British (African): Senior Lecturer in Law**].

This involved acting in ways that she believed were consistent with a (white) British prototypical standard and etiquette:

“*I have more opportunities to pull a side of myself forward, it all comes down to commonality—it is explained through commonality (on the positive side), when it's a positive interaction, I bring forward the elements that are the same as the person I am relating with, whether that's gender, race, religion or nationality”* (**Participant 12—British Born Nigerian: Retail Business Owner**).

This above quote, corroborates findings from Ogbu (2004) demonstrating that Black (African/Caribbean ethnic) individuals, trying to assimilate to the workplace culture, may need to distance themselves from their ethnic culture and dialect and speak and behave primarily in ways that signals prototypically white British etiquette.

#### Flexing identity to build professional relationships and for professional advancement

A number of other participants also mentioned the ways in which they instrumentally flex identity when presented with the opportunity to do so, to gain benefits for themselves:

“*Being an African woman can definitely be advantageous, it has gotten me jobs […] For example, in a meeting with an African woman, I push that identity forward, it absolutely works in my favor, as we are both African women”* (**Participant 12—British Born Nigerian: Retail Business Owner**).

Alternatively, their ability to leverage a part of their identity may enable them to achieve more in their work:

“*When working with families from AAC backgrounds it is often (rightly) assumed that I have a greater understanding of the ethno-cultural dissonance they may be experiencing. At this point I use aspects of my ethnic identity to navigate the situation in terms of relating more deeply. It acts as a bridge to forming greater connectedness.”* (**Participant 15—Black British Caribbean: Educational Psychologist**).

Participant 21, a Senior Lecturer in Law who identifies as Black British (African), explained how she emphasizes “*my race or aspects of my race”* to connect with [AAC] colleagues who are sometimes “*more senior than I am, within other departments, via the networks that we are able to form.”* In this example highlighting or flexing AA or C identity was a useful strategy for this participant to build relationships with other AAC colleagues. This also highlights that the women may flex identity toward their racio-ethnic identity as well as away from it, determined by what aspect of identity needs to be made dominant within the given context. One participant highlights this by explaining how she is able to flex her ethnic identity, in a way that proves resourceful when she undertakes travel for work, she states:

“*I often travel for work. I find that I am able to relate and engage with our international business partners with greater ease, on the basis of my ethnic identity. […] An Indian international business partner once told me, “you are welcome (he did not say this to my colleague), as we do not see you as the colonizer,” this statement was made in front of my white colleague.”* [**Participant 28—Nigerian (British Born) Senior Lecturer**].

This comment, highlights that one's identity (and therefore identity experiences) are fluid and transient within social context(s). It highlights the versatility that comes from having an identity that comprises aspects of both the Global North and the Global South, it means there is a possibility for one to “belong” in both contexts. This is often not the case for a large majority of white British individuals, who have lived in the UK for their entire lives. In a similar example, Participant 16 described how she flexes identity for the purpose of gaining influence within organizational settings. Participant 16 recognizes that the opportunity that she was able to create for herself was due to the efficacy of the “Black woman” identity within situations concerning matters of race and diversity in organizational settings, and that this ultimately facilitated her professional advancement:

“*I use my ethnic and racial identity to gain advantage but only when it is used to help other [AAC] people, I recently contacted my university's AAC network and addressed the chairs. […] I told them, let me help develop the network, given my experiences and I am a Black Caribbean woman, they bit my hand off for it*.^2^” (**Participant 16—Black Caribbean—I am not British, I don't even know what that means, even though I was born here: Lecturer**).

These results highlight how having undervalued or marginalized aspects of identity may not always result in subjugation or disadvantage. This seems to be partially due to the distinctiveness of a minoritized identity, which may lead to unique opportunities to effectively leverage facets of ones' identity, within particular social contexts and interactions.

### Theme 2: The role of specific stereotypes

#### Intersectionality, stereotypes and identity flexing

Women have been perceived as not possessing sufficient agentic characteristics to be leaders, managers or change agents. Yet this stereotype is based predominantly on white Western women. Rosette et al. (2016) refers to “intersectional effects” of raced and gendered stereotypes, to elucidate the nuances of stereotype content when considered at the intersection of two or more facets of identity. This means that when we consider AAC women we find that the intersectional stereotype narratives (i.e., the interactive narratives of race-based *and* gendered stereotypes), of Black African/Caribbean women as confident, dominant, and assertive and Asian women as competent, yet passive means that AAC women are at times able to exhibit agentic traits such as strength, competence, dominance, outspokenness and receive less backlash for displaying these behaviors (Rosette et al., 2016). These group-based stereotypes were some of the conditions that women claimed play a role, in the ways they flexed an identity to navigate a situation. For example, one participant described how pushing her AAC woman identity forward, allowed her to speak on matters in a somewhat unfiltered or unaffected way:

“*I guess the place that I am at now, is that my Blackness [African identity] gives me the rite of passage to say whatever I have to say”* (**Participant 12—British Born Nigerian: Retail Business Owner**).

In a similar example, a second participant illustrated that she was consciously utilizing the “docile” stereotype, which is often unfairly attributed to women of Asian descent, in-order to achieve a desired outcome. She states:

“*People have an expectation that I am supposed to be more docile and not respond in certain circumstances. I am not told in an obvious way, […] sometimes I will give colleagues what they expect, when it is going to work in my favor, for instance I am able to work away in the background, conceal my successes and not be challenged about this, due to the assumptions that others make about me”* (**Participant 8—British Indo (Goa) Portuguese: Technical Consultant**).

For many Asian women gender and racio-ethnicity intersect to produce a particularly fraught position for them. Asian women are likely to receive backlash or be harassed when they violate the prevailing prescriptive stereotype that is held of them by white colleagues (Rudman and Phelan, 2008; Rosette et al., 2016). Where Asian women are expected to be *competent, yet docile*, those who disturb this narrative, are likely to be deemed threatening or rude (Berdahl and Min, 2012). In turn, Participant 8 expresses the way in which she might flex her identity and endorse a stereotype, to be afforded the resource of time and space to conduct work geared at ensuring her personal success.

Participant 8 was not alone. Other women spoke of ways in which their particular identity provided them with a speaking position. Participant 1 mentions leaning into her identity as a British woman with African ethnicity, and how this allows her to be unapologetic about speaking out and not needing to filter her thoughts at certain times:

“*I emphasize my racial and ethnic identity, I use it to explain my need to differ at times and it gives me the freedom to be unapologetically different if I want to be, with this you get to say what you feel with very little resistance***”** (**Participant 1—Mixed Black and Asian Caribbean: Human Resources Manager**).

In the above quotes we see a contrast between what white British women *get* to do and what AAC British women *get* to do based on the nature of the stereotypes that women are likely to be subjected to. Often for white women who speak out and say what they feel, they are referred to as “brash” or “bossy” (Rosette et al., 2016), whereas the same was not the case for the previous (AAC woman) interview participant. In other words, one's particular experiences at the intersection of aspects of identity informs which ways they are able to use their identity to influence an outcome or exploit an opportunity.

In accordance with Rosette et al. (2016) the stereotypic content of AAC women and white women clearly uncover that there are discrete stereotypes related to each group and discrete consequences for women from each subgroup of women (i.e., Black /African, Asian and white women). Simply stated, Black (African/Caribbean ethnicity) women are perceived as being dominant but not competent (Donovan, 2011). Asian American women are perceived as being competent but passive (Castro and Collins, 2021). White Western women are perceived as primarily communal without being seen as particularly dominant or excessively competent (Rosette et al., 2016). Consequently, Black women are the least likely to suffer agentic penalties, then Asian American women and finally white women are most likely to suffer agentic penalties. The pattern is reversed for agentic deficiencies (Rosette et al., 2016).

“*Yes, there are definite privileges to being Black. They regularly assume that you are speaking for your entire race. I think this is the fatal error. I speak for myself actually and from my experiences. However, as a Black woman, I get to stop them in their tracks and assert myself and say, listen I don't have expertise in that…so ask another Black person. This tells them categorically what my stance is, where my boundaries and limitations lie. It could be an advantage because I don't see many women being able to say exactly what they feel”* (**Participant 16—Black Caribbean—I am not British, I don't even know what that means, even though I was born here: Lecturer**).

Participant 16 provides insight into her experience of being able to assert herself and at times say what she feels, particularly in times when assumptions are being made about who she is and what she might think about a situation. Participant 16's quote also highlights her recognition of being able to express herself in a way that is distinct to other women, possibly due to the prevailing stereotypes that are attributed to Black women, as domineering or outspoken. This suggests that the content of a social stereotype toward Asian ethnic women or Black (African/Caribbean) ethnic women differs from stereotypes related to women generally, as this stereotype is largely based on the stereotypes attributed to white Western women. Generally, therefore, this demonstrates the role of a particular intersectional stereotype toward specific groups of women, compared to other groups, in-order to capture the specific nuances of stereotypes and how these inform women's experiences. According to Petsko et al. (2022, p. 765) intersectional stereotyping recognizes that perceivers can stereotype Black women, for example, not as Black more generally or as women more generally, but as Black women specifically.

Flexing toward certain types of agentic behavior could be a viable strategy for AAC women and could lead to advancement. Participant 6 focused on how emphasizing her agency and competence, allowed her to navigate situations more readily:

“*I have felt the need to dig my heels in at times, if you don't, they can often see you as a push over. I suppose I can dig my heels in, with little consequence, they see me as the outspoken ethnic one! I don't know if white women get to do this?”* (**Participant 6—British-Asian: Social Care Manager**).

These accounts suggest that despite an understanding that stereotypes of being competent but passive (for Asian women), or confident yet aggressive [for African and (Black) Caribbean women] are indeed negative, such stereotypes may have afforded AAC women the opportunity to express their views and behave in agentic ways. Therefore, the backlash against agency that women experience in the workplace may be based largely on such stereotypes (and experiences) of white Western women. This once again speaks to intersectional invisibility, producing a mix of advantages and disadvantages rooted in the notion that one's unique intersectional identity means that often, others are not able “pin them down” with a particular set of biased stereotypes.

### Theme 3: Context specific opportunities

Like Theme 1 which describes the more general benefits of identity flexing, Theme 3 highlights particular benefits or contexts that the participants were able to leverage opportunities. As Purdie-Vaughns and Eibach's (2008) theory on intersectional invisibility suggests, intersecting non-prototypicality can also generate certain adverse implications, and so these opportunities are not an unequivocal reflection of “power” in the traditional sense of that term. Such opportunities are characterized by the individuals' need to pull on more than one facet of their identity within work environments, and therefore they are considered context specific opportunities reserved for AAC women (and possibly other individuals whose intersectional identity comprises elements of under-valued group identity categories).

#### Class, nationality and voice

Participants spoke about how leveraging context-specific opportunities pertained to several of their social identity categories simultaneously and how the context plays a role in allowing this (e.g., gender and ethnicity or nationality, voice, and racio-ethnicity). They also explained how they consciously get to choose the way in which they accentuate intersectional facets of their identity, based on certain identity facets within the particular context, and on a situation-by-situation basis. The following quote highlights the use of British nationality and native accented speaking voice as a proxy for class and as a source of empowerment within the workplace. This reveals that the UK context and possibly being British born, are important to allow this participant to flex identity in this way, due to her accent proxying belonging within the UK context. The work of Rodriguez et al. (2016), explicates that there is an intersectional approach that focuses on subjectivities and investigates identity intersections to better understand the nature and implications of inequalities experienced by individuals and groups (Crenshaw, 1991). This subjective approach to intersectionality is prevalent within this study's results, as we are able to specifically regard the intersectional costs and benefits of strategically flexing identity. As a result, we see that Participant 28 was able to flex aspects of her identity by accentuating her behavior, language, and British culture in accordance with the dominant British workplace culture:

“*I am very clued up on British culture and history/heritage. I also speak with a very clear English accent. My accent certainly signals my Britishness and my class, it means at times they must listen to me, whether they like it or not, it means I can also correct white people on matters of Britishness”* [**Participant 28—Nigerian (British Born) Senior Lecturer**].

This flexing allowed her to avoid being labeled with many of the negative stereotypes that AAC women face, as well as allowing her to control her own identity narrative.

#### Ethnicity and gender

Perceived contextually, opportunities, were also leveraged through the flexing of ethnicity and gender identity. Participants mentioned that their ethnic identity could be used as a resource to interrogate issues of race and gender inequality more freely, in particular social spaces, when the opportunity to do this occurred. The participant below who was natively born in the African context and went through the naturalization process as an adult, to become a British citizen, utilizes this strategy to get her colleagues onside. This participant mentions working in a UK Higher Education Institution, where there is a substantial white British majority, she states:

“*In shared staff areas, I have cheekily used my ethnic identity to pretend not to understand boundaries, this allowed me to talk away and explain my opinions about the gender pay gap and race pay gap. I simply told them, ‘I do not understand how the race pay gap can be justified because I come from Africa where we are all black, so such a thing can't exist'. They get it and by the end they are equally angry about it and are vowing to try and do something about it”* (**Participant 21—Black British (African): Senior Lecturer in Law**).

Participants did not trivialize the contextual potency of being an AAC British woman, within the UK context. It was noted by the majority of women that their particular intersectional identity “*is not common within my organization*,” which allowed them to consider their intersectional identity valuable and a unique resource. One particularly relevant line of theorizing that falls within the realm of [various lines of theory and research on] intersectionality (Purdie-Vaughns and Eibach, 2008) is that if an individual is considered as a non-prototypical member of their identity groups, they will experience *intersectional invisibility*. By this, it is meant that these individuals will not be seen as full members of their constituent groups. Intersectional invisibility helps to form a foundation for exploring the notion that AAC women may stumble upon context specific opportunities to *flex* their identity in ways that others cannot. Taken together, this should be deemed generally advantageous as it could allow the women to avoid adverse consequences, or leverage opportunities due to flexing a precise and unique aspect of their identity as a British, AAC women.

Participant 28 asserts:

“*I am Black, Igbo, Nigerian, African, British and a woman, this is my pride and my advantage, as I get to make any of these identities salient, whenever I need them, I have to celebrate this.”* (**Participant 28—Nigerian (British Born): Senior Lecturer**).

The women at times, also regarded their particular intersectional identity as advantageous and/or resourceful in the workplace:

“*I am female, I am brown or black (at work, where others are not), so these two things will potentially open doors for you in some ways, so why wouldn't you use it”* (**Participant 4 - Mixed Black and Indian Caribbean: Health Administrator**).

### Theme 4: The costs of identity flexing

Another prevailing theme that emerged from the interviews, involves the notion of having to grapple sometimes with negative emotions and the feeling of not being one's authentic self, resulting from needing to flex identity in a strategic way. Although there are many advantages of possessing a unique, and less prevalent (within the UK context) identity, such as being a British AAC woman, there are also costs to engaging in identity flexing. Whilst other groups may utilize their identity when flexing identity in a resourceful way, it is apparent from our results that AAC women feel they need to engage with this phenomenon on a regular basis in their work environments. This is likely to lead to feelings of not being able to be one's authentic self at work, as the stimulus for one to flex identity is often, due to their particular identity being put into question.

#### Inauthenticity

Indeed, individuals who have to regularly flex or highlight aspects of their identity over another may be subject to negative costs. This may include having to engage in greater amounts of emotional and psychological labor or feeling that they are not able to be their true selves. Thus, it may likely be difficult for AAC women to manage their identity and multicultural experiences, whilst being conscious of the pressure of having to frequently negotiate the various features of their identity. This in turn is likely to result in poorer psychological health:

“*Sometimes when I change from one situation to another, I reflect and say, okay I was this person for that person, and now I am being this person for someone else, so great! I get to choose, but who am I then when I am in the presence of both of these people? [..] this can get tiring.”* (**Participant 20—African: Mental Health Nurse**).

Individuals who regularly engage in identity flexing strategies may also be perceived as insincere by those individuals who may be less likely to regularly flex identity, such as white British men. This could lead to individuals having to grapple with an internal sense of feeling inauthentic:

“*Sometimes it can feel as though the more identities you try on, the more blurred the idea of an ‘authentic' identity becomes […] When I present more of typically white British traits it can make me feel inauthentic to a degree, but I have come to consider myself as a bit of a social chameleon, so I guess that is an identity in itself.”* (**Participant 15 – Black British Caribbean: Educational Psychologist**).

The comments from Participant 15 possesses something of an undercurrent of one's personal struggles due to the colonial legacy of not fitting in, within the context that one finds themselves. We say this, as the waves of migration that ensued throughout post-colonial eras, are to some extent a reason for the substantial African, Asian and Caribbean populations that you are likely to find in the UK. This helps to evidence the dynamic interplay between one's identity and the underpinning instituted systems of domination at the societal level in perpetuating subjectivities and intersectional difference at the organizational and individual level (Dhamoon, 2011).

#### Less social direction

The interviews also revealed that AAC women may receive less social direction on what it means to belong to their identity groups, specifically when considering the experiences that occur at the intersection of gender and racio-ethnicity. Moreover, the people they interact with may also have little guidance on what it means for a person to possess their specific intersectional identity. Participant 28 who identifies as “Nigerian (British Born), states that she was asked by a colleague who was natively born in West Africa, but had since migrated to the UK, “*Do you prefer I treat you more like a white person, or should I just treat you like a proper African, born and raised like me?”*. This evidences the increased identity tensions that British AAC women may need to navigate, leading to feeling uncertain about who they are, feeling inauthentic as well as coming to terms with the high possibility that they will be misunderstood by others. **Participant 29 a Business Consultant**, who identifies as a **Black African Woman—British Born**, alludes to this, she states:

“*Other Black people expect you to know a lot about being Black or African and then white people want you to know lots about being British, but none seem to really understand that there is this intersectional space in the middle of British-African....to be African and British is not a performance, it just is what it is.”*

From the quote above we can see that a possible cost of flexing from one aspect of identity to another is a less defined notion of what it means to be a British AAC woman. It is important to recognize that this evidence illustrates *potential* costs that can emerge from identity flexing—they are not inherent or universal costs. In this way, we hope to better clarify for readers that while the data indicates some relatively consistent themes around potential costs, there is inevitably some degree of variability in how and to what extent any two individuals (or a single individual across different situations) will incur any potential costs when engaging in identity flexing. At the same time, much of the data does indicate that AAC professional women are not just coping with or being *reactive* to experienced struggles and disadvantage. They are also *proactively* pursuing positive outcomes through strategic identity flexing.

## Discussion

To our knowledge, our study is one of very few that has focused on how professional AAC women *flex* facets of identity in ways that could be deemed strategic or constructive. We explored whether such flexing was useful in navigating a situation or exploiting a particular opportunity. We also examined whether there were certain costs associated with identity flexing, that is whether the notion of identity flexing, causes us to question *who* needs to adapt? The study is not able to make inferences concerning whether white British individuals (e.g., white men or women) need to flex identity in a similar way, as the study focuses on British AAC professional women, although none of our participants spoke of their white British colleagues also using flexing strategies. For AAC women, because they are subject to intersectional invisibility, often having to adapt their behavior to fit the prototypical behavior within the context, this means that they feel the *need* to regularly adapt behavior, language, accent, and/or appearance.

Our analyses demonstrated that AAC women's experiences of identity flexing were underpinned by a particular type of advantage. At the same time, this particular group of women's experiences also showed that there can be costs to engaging in strategic identity flexing, such as having to perform increased amounts of emotional labor, or the inability to be authentic in the workplace. This interplay between advantage and disadvantage highlights, in accordance with Bottomley (1991), that the workplace identity experiences of AAC women are the dialectal interplay of self-identification, identification by others, the result of structural forces, underpinning systems of difference and domination. Thus, identity flexing in the work context is a very interactive process, which occurs against a backdrop of privilege and penalty, reproduced and resisted in contextual and relational ways (Dhamoon, 2011). The appeal of identity flexing for AAC women and other minority groups is that the application of these types of strategies concerns the dynamics of power (see Morton, 2013). This means that, when the flexing of an identity occurs it leads to a sense of empowerment, as the individual can strategically endorse or reject valued or undervalued aspects of identity in a way that proves advantageous to them (Opara et al., 2020).

### Advantages and disadvantages of identity flexing

Study participants highlighted the forms of contextual advantage they believe they have, typified by versatility, flexibility, and empowerment. Thus, identity flexing can help British AAC women develop *effective* identity management strategies as it acts as a mechanism for influencing moments of self-representation. It can at times enable women to temporarily control their own identity narrative, and craft out experiences of empowerment wherein they get to exploit their versatility. Ultimately, this may help in achieving certain aims such as accessing equal rights, generating certain opportunities, and evoking a sense of empowerment, but this sense of empowerment is not without costs. For instance, many of our study participants described grappling with the disadvantages of identity flexing. Indeed, some believed that it was necessary to modify their behavior, language, accent, or physical appearance to navigate organizational and social contexts, while concurrently being aware of the negative impacts on wellbeing and the increased amount of emotional labor, and feelings of inauthenticity that they had to contend with.

#### Emotional labor

Increased emotional labor can be understood within numerous facets of psychological health and wellbeing, in that, for these women “fitting in” is intertwined with being accepted. Participants specifically expressed that the pressure to fit in can be tiring and if this perceived pressure persisted then this led to having to manage negative emotions. The need for AAC women to have to manage negative emotions means they are more likely than their white British colleagues, to be engaging in emotional labor (see Mauno et al., 2016). Notwithstanding, the interview accounts that we collected were nuanced, as these experiences of engaging in increased emotional labor were considered alongside the resourcefulness of identity flexing for AAC women and individuals. There is research that suggests that adopting identity management strategies, such as flexing, may reduce feelings of emotional exhaustion, increase authenticity and has potential advantages for organizational health and wellbeing (Shih et al., 2010; Mauno et al., 2016). From our research we can deduce that the possible health benefits are due to the positive emotions that can be associated with being able to control one's own identity narrative to allow for greater consistency between one's self concept and how perceivers are seeing the individual. This does not mean that it is the *responsibility* of AAC women to seem more similar or connected with colleagues of other ethnicities or genders. AAC women should not have to carry the weight of managing colleagues' unfair perceptions of them. However, the reality is that they do this on a day-to-day basis.

Nevertheless, where one experiences positive emotions that can be associated with being able to control one's own identity narrative, it means the individual is more likely to feel that, to some extent, they are able to be their authentic self in the workplace. We say this as positive feelings have been associated with improved health status and thus, increased wellbeing (Fredrickson and Losada, 2005; Sojo et al., 2016).

#### Inauthentic experiences

The pervasiveness of inauthentic experiences occurs as a theme within the study results. Authenticity has been defined as the genuine expression of self, inclusive of emotion, with little prompting (Ashforth and Tomiuk, 2000), and researchers have found that authenticity and authentic experiences play an important role in reducing levels of anxiety and psychological burnout. Our results about inauthentic experiences are largely in line with other intersectional study results (see Dickens and Chavez, 2018; Doldor and Atewologun, 2020) illustrating that professional AAC women are seldom afforded the freedom to be their authentic selves in the workplace (Opara et al., 2020). In the face of this reality, it becomes more difficult for AAC women to manage their identity and organizational experiences, whilst concurrently being conscious of the pressure and stress of having to frequently negotiate identity to highlight similarities between their work colleagues and downplay the differences. Nevertheless, there were several women who asserted that they were being their authentic selves, in that the aspect of identity that they “tried on” belonged to them and was authentically their own. The idea that the ability to flex identity and “fit in” as a sort of versatile social chameleon was also suggested to be an identity in itself. This line of thinking helps to debunk the notion of identity being linear, static, and prototypical, but rather identity might also be fluid, intersectional, functional, and non-prescriptive (Opara et al., 2020).

### The importance of recognizing this mix of advantages and disadvantages

Taking the advantages and disadvantages of identity flexing together, we assert that some of the unique advantages of employing identity flexing strategies are not necessarily a viable, long-term, organizational level approach to ensuring positive workplace experiences for all individuals, there are also costs embedded within implementation of identity flexing strategies (Cole, 2009; Opara et al., 2020). This suggests that cultivating a work environment whereby individual differences are accepted at least and celebrated at most will allow employees to engage in open and honest discourse about what it really means to belong to their particular social identity groups. Resorting to strategies like flexing could suggest that at times racio-ethnicity and gender identity is being put into question. For instance, Alvesson and Sveningsson (2003) assert that individuals and groups strive for belongingness and acceptance. Whereas not being accepted fully, makes people unable to portray themselves in an authentic way.

Overall, the study results regarding the potential advantages and disadvantages of identity flexing align with previous research (Purdie-Vaughns and Eibach, 2008; Shih et al., 2013; see Opara et al., 2020), suggesting that the long-term use of identity flexing strategies can cause a volatile sense of self, and this volatile self can be linked to poor psychological wellbeing (Shih et al., 2013). In a similar vein, research suggests that if an AAC woman chooses to assimilate to her prototypically white British workplace culture, in efforts to obtain certain benefits or avoid disadvantage in that context, on transitioning from the organizational context to one wherein her AAC ethnic identity has more value (e.g., home), she may find that displaying the same type of behavior triggers feelings of betrayal or a lack of loyalty to her respective AAC community, be that an Indian community, West African community or any other community represented within AAC ethnicities. Yet if she decides *not* to flex her identity toward prototypically white British behaviors at work, then she may concurrently feel a sense of connectedness with her fellow AAC colleagues but incur a cost toward professional advancement (Rodriguez and Scurry, 2019).

It is important to acknowledge that having to grapple with, feelings of inauthenticity and a blurring of what it *actually* means to be an AAC woman, formed just a part of the women's richer workplace experiences. This is important as the true nature of AAC women's organizational experiences is not yet comprehensively understood. Furthermore, where the experiences of this particular group of women are not understood, we risk further constraining them into a prevailing narrative of what it means to be an AAC professional women. In turn this increases the risk of reinforcing discriminatory views based on racio-ethnicity and gender (Rodriguez and Scurry, 2019; Opara et al., 2020).

## Limitations and future research

This study advances knowledge regarding experiences and effects of strategic identity flexing at the intersection of racio-ethnic identity and gender identity, it demonstrates that although identity flexing can be deemed advantageous, it is occurring against a backdrop of privilege and penalty, as AAC women's identity is being put into question; so implicit to identity flexing are the costs of having to modify or manage one's identity in this way. The mix of both advantage and disadvantage limits the utility of identity flexing as a sustainable approach to accepting and possibly commending diversity and difference in work organizations (Cole, 2009). This has likely implications for retention, as the ability to retain employees, requires managers to build a workplace that embraces diversity, be that cognitive diversity, values diversity, cultural and demographic diversity or otherwise (Özbilgin and Tatli, 2008). These are important results, which help us to move scholarship beyond the parameters of what we already know about this group of women and their workplace experiences, into an *uncharted* terrain. This is a valuable contribution to knowledge, which we believe should not be adulterated, glazed over, or minimized.

A limitation of this study is the lack of attention to whether AAC professional men are also able to mobilize intersectional identity creatively to leverage opportunities and negotiate challenges. Scholars within the field might wish to investigate whether AAC men (and other groups) are able to leverage opportunities in agentic ways, as we know that there may be instances when two or more undervalued facets of identity intersect to provide AAC women professionals with an advantage at work, but whether this is true for one undervalued facets of identity intersection (as is the case for AAC professional men and other groups, e.g., white women), is yet to be known. This is important because advantageous workplace identity experiences can positively affect other areas of one's life, so implications may likely extend beyond the social frontiers of the work organization. Future research could also deal with how organizations foster openness when it comes to diversity as well as considering which types of flexing might (not) occur in particular instances, as advantages and disadvantages could be experienced differently by different people/groups.

Another study limitation concerns the ethnic identity labels within this study. Participants self-ascribed their racial/ethnic identity, which meant that very few women solely identified as “African” or “Asian” with participants identifying as “*British-Nigerian, Black British, Black Caribbean, Igbo Indigene, Indo-Portuguese, Indian-Jamaican, Mixed Black–Caribbean and White–British, South Asian Sri Lankan, and Nigerian*. One participant emphasized that she was British born, but then rejected it as an identity category that she chooses to regard, instead identifying as: “*Black Caribbean, I am not British even though I was born here.”* We perceive the discrepancy between how individuals self-identified and the identity categories that we refer to in this paper as a limitation of the study. Although the African, Asian, and Caribbean might capture something of the ethnic and cultural background of the women, these labels do not do enough to appropriately reflect the interpretivist underpinnings of the interview participants' self-ascribed ethnic identity. Furthermore, the ethnic identity labels do not completely communicate the vast and distinct nature of ethnicities represented within regional categories such as “African,” “Asian” or “Caribbean,” and this means some interpretation is likely to get “lost” because of this. For example, we understand that the term “Asian” encompasses China and India—the two most populous countries in Asia and globally (comprising 35% of the global population) (United Nations, 2017). Similarly, “African” contains Nigeria and Ethiopia the two most populous African countries, having a combined population of over 320 million inhabitants (United Nations, 2017). Future research should explore experiences of identity flexing, whilst disaggregating the sample to regard ethnic group identity within regional context, rather than a broad and often non-productive regional identity. Nevertheless, this paper does well to move away from the crude and unclear “BAME” label, which we previously explained, combines all “non-white” British individuals plus some white ethnic minorities. BAME as a category is unproductive, as it disguises the lived experiences of individual racial/ethnic groups represented within it (Brunsma and Rockquemore, 2001; Rockquemore et al., 2009). This paper also moves away from the out-dated and overly-simplistic “Black–white” socially constructed, contrast race labels, of which many are yet to do. The categories constrain bi-racial and multi-racial individuals within an outdated monoracial scale of race and ethnicity and incorrectly group the experiences of all Black and all white people, irrespective of location or experience, e.g., North America, Europe, Africa (Brunsma and Rockquemore, 2001).

## Conclusion

In this study, we sought to provide a better understanding of the identity flexing strategies that British professional AAC women may adopt in UK work organizations. In contextualizing the workplace experiences of British AAC women, at the intersection of gender identity and ethnic identity, in relation to identity flexing strategies the study shows that the experiences of this group should not be pigeon-holed to only reflect those experiences of disadvantage such as discrimination, stigmatization and negative stereotyping. Throughout the paper we alluded to the need for nuance in the investigation of AAC women's workplace experiences, in-order to challenge dominant and restricting narratives, that present AAC women's organizational experiences as wholly oppressive. The study's results support the results from the Opara et al. (2020) study, that discrimination and disadvantage that AAC women face in work organizations do not represent the *whole* of British AAC women's experiences. There is a need to also consider when experiences are likely to be deemed advantageous and could become a potential resource. It is important that we understand AAC women's experiences of advantage. This will involve recognizing that alongside experiences of disadvantage and marginalization, AAC women's strengths, knowledge, creativity, and insights, may also allow them to proactively leverage opportunities. We have contributed to this nuanced investigation by highlighting how opportunity, advantage and disadvantage are contextual phenomena, which are produced and contested in relational ways. There are key areas to focus on to help progress the discussion and understanding of AAC women's organizational experiences. These key areas include further exploration of social context, in producing and reproducing the vast experiences that AAC women have. In addition, further exploring AAC women's role in shaping their experiences, be that through identity flexing strategies or in other ways. Finally, further research should investigate whether the same costs and benefits of employing identity flexing strategies emerge for other populations, e.g., bi-racial individuals, working class individuals, invisible minorities, and AAC men.

## Data availability statement

The raw data supporting the conclusions of this article will be made available by the authors, without undue reservation.

## Ethics statement

The studies involving human participants were reviewed and approved by Nick Moberley University of Exeter Ethics Committee. The patients/participants provided their written informed consent to participate in this study.

## Author contributions

VO, MR, and RS conceived and planned the study and contributed to the interpretation of the results. VO carried out the interviews and took the lead in writing the manuscript. VO and RS contributed to sample preparation. VO, CB, MR, and RS provided critical, helped shape the research, and analysis and manuscript. All authors contributed to the article and approved the submitted version.

## Footnotes

^1^For example, in the UK, the demographic group that performs worst educationally is white working-class boys (HEPI, 2020): https://www.hepi.ac.uk/2016/05/12/new-hepi-report-reveals-educational-underachievement-young-men-higher-education-calls-sector-tackle-problem/.

^2^Explanation: “to bite someone's hand off” is a colloquial expression, which means to be in such a hurry to take what they are offering, that you snatch it out of a person's hand, like a dog who is so eager to get a treat that he almost bites your hand off for it.
