# Supplementary material for: “Fitting in whilst standing out”: Identity flexing strategies of professional British women of African, Asian, and Caribbean ethnicities
Source: Front Sociol. 2023 Mar 23;8:820975. doi: 10.3389/fsoc.2023.820975 (PMC10076833; doi:10.3389/fsoc.2023.820975)
Supplement: Supplementary file 1 [file Data_Sheet_1.docx]

**Appendices: Appendix 1**

**Interview Script**

**Demographic Information**

This section aims to collect demographic information so we can get a sense of who you are and ensure we get a representative sample – this information will not be used to identify you as an individual.

Age (please specify):

Gender (i.e. male, female, other - please specify):

What is your highest qualification? (i.e. Undergraduate, postgraduate degree, diploma)

What sector do you work in (i.e. Agriculture, Education, Health)?

What is your job role?

How many years have you been in your current job role? (please specify):

Is your current job role part-time or full-time? (please specify):

Do you consider yourself to be an African, Asian, Caribbean and / or Mixed - British woman? (Please underline yes or no)

YES                                                                 NO

Please describe your particular identity below (e.g. British-Bangladeshi; Mixed Black Caribbean and White British, etc.)

**Study Questions**

*We all have multiple aspects to our identity, we’re going to start by looking at different aspects of who you are at work.*

*Who you are*

1.             Which of your group identities (e.g. ethnicity/race, gender, nationality) are most important to you when at home?

*2.*             Which of your group identities (e.g. ethnicity/race, gender, nationality) are most important within your community?

*Who you are at work (identity imposition)*

*3.*          How do other people see you at work?

*4.*             When thinking of your work colleagues, which of your group identities (e.g. ethnicity/race, gender, nationality) do you think are most obvious (salient) at work?

*5.*             When thinking of your work colleagues, which of your group identities (ethnicity/race, gender, nationality) have a bearing on how you act / carry yourself at work (e.g., your behaviours around colleagues, your dress/appearance, etc.)?

*6.*              Do you ever feel that you are told what the proper behaviour / attitude for you is? (e.g. both positive and negative behaviours / attitudes)

*7.*             Have you ever had any experience of others making assumptions about you (e.g. ethnicity/race, gender and / or nationality) because of how they see you?

*8.*             Can these assumptions be advantageous?

9*.*          Have you ever had any experience of others seeing you in terms of particular identities, that is different from being discriminated against?

*Strategies to navigate identities at work (strategic identity flexing)*

This section is going to look at how you deal with/cope with these identity experiences, what strategies or coping mechanisms you might use.

*10.*      How do you deal with these experiences?

*11.*    How did you respond – at the time or later?

12. Do you ever seek to make certain identities more obvious in the workplace?

*13.*     Is it ever possible to modify your behaviour or play-up certain identities to capitalize on the level of advantage and opportunity?

*14.*  Do you modify your behaviour or play down certain identities to lessen the load of disadvantage? If so how

*15.* Have you ever used aspects of your ethnicity/ race and nationality to gain an advantage / navigate a situation / strengthen a relationship, etc…?

*16.* Do you modify your behaviour when you are unsure of what behaviours / treatment to expect from another person e.g. a colleague, a neighbour, etc?

*Outcomes of navigating identities at work (Well-being)*

In the final section, we’d like to look at some of the outcomes or impacts that these experiences might have had.

*17.*          Do you feel you can bring your “whole self” to work?

*18.*          How did it make you feel in terms of your ability to be authentic?

*19.*         How did it make you feel in terms of the level of stress that you experienced?

*20.*    We’ve talked about how that (those) experience(s) made you feel in the moment, what about the longer term, what impact do you think they may have in the future?

21. What impact do you think they may have on you?

**Appendix 2**

| **Main Probes:** | 1. How did you respond to this experience/treatment/event? 2. Why do you think you responded in this way? 3. How did it make you feel? 4. What were your thoughts about this experience/treatment/event? 5. What did you do about it? 6. Can you provide an example? 7. What happened next? 8. How did colleagues respond? 9. How did management respond? | | 1. Why do you say this? 2. How did that make you feel? 3. Why exactly is this a good/bad thing/idea? 4. Is this a problem to you? 5. How so? 6. How did you respond? 7. Can you provide an example of this? |
| --- | --- | --- | --- |
|  | |  |  |
